# Supplementary material for: SARS-CoV-2 neutralizing antibody specificities differ dramatically between recently infected infants and immune-imprinted individuals
Source: bioRxiv. 2025 Jan 20:2025.01.17.633612. Preprint. [Version 1] doi: 10.1101/2025.01.17.633612 (PMC11785066; doi:10.1101/2025.01.17.633612)
Supplement: 1 [file NIHPP2025.01.17.633612V1-supplement-1.pdf]

bioRxiv preprint doi: https://doi.org/10.1101/2025.01.17.633612; this version posted January 20, 2025. The copyright holder for this preprint (which was not certified by peer review) is the author/funder, who has granted bioRxiv a license to display the preprint in perpetuity. It is made available under aCC-BY 4.0 International license.

Supplementary Figures

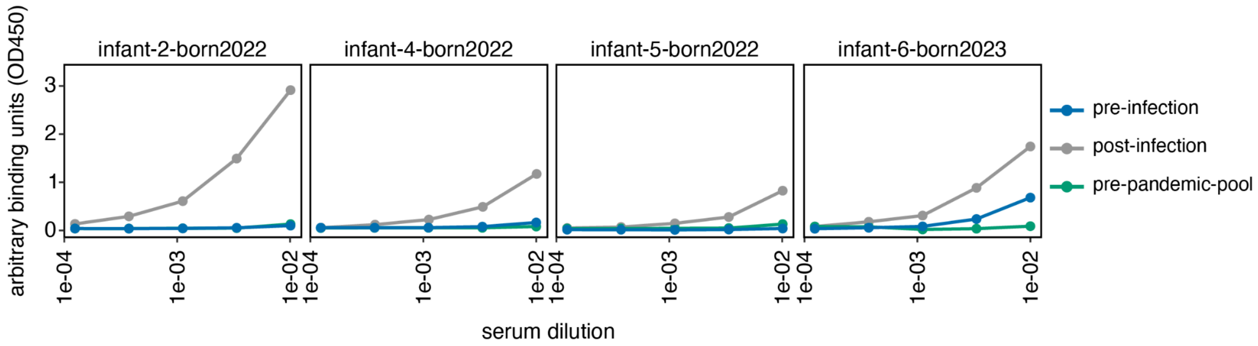

Supplementary Figure 1. Binding of the infant sera to RBD-coated ELISA plates pre- and post-infection with a XBB* variant

Sera binding to XBB.1.5 RBD-coated plates measured using enzyme-linked immunosorbent assay (ELISA). The low RBD binding activity in the sera collected prior to infection indicates maternal SARS-CoV-2 antibodies had decayed to very low levels prior to infection. As a negative control, we binding is also shown for a pool of human sera collected prior to the beginning of the COVID-19 pandemic. Only four of the 6 infants in the *primary XBB* infection infant sera* group had pre-infection sera available. The pre-infection sera was collected 11-46 days before infection and therefore at the time of infection maternal antibodies may have decayed further. See **Table S1** for details on sera collection times.

bioRxiv preprint doi: https://doi.org/10.1101/2025.01.17.633612; this version posted January 20, 2025. The copyright holder for this preprint (which was not certified by peer review) is the author/funder, who has granted bioRxiv a license to display the preprint in perpetuity. It is made available under aCC-BY 4.0 International license.

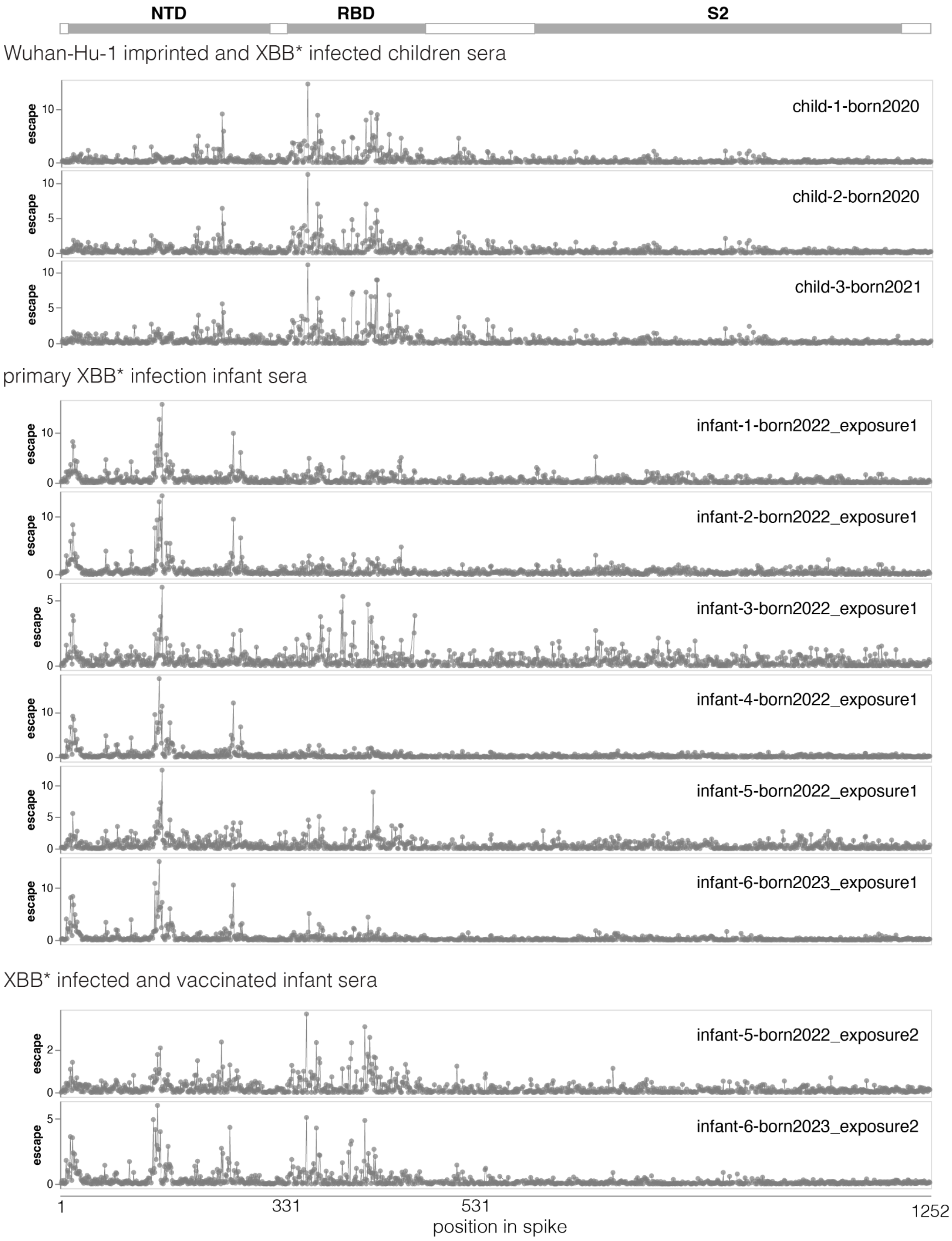

Supplementary Figure 2. Deep mutational scanning escape maps for individual sera

These plots are similar to those in **Fig. 2A** except they show escape for the individual sera in each set rather than the average across all sera in each set. This figure only shows the plots for the three sera sets that were newly characterized in this study; the plots for the *Wuhan-Hu-1-imprinted and XBB* infected adult sera* have been previously published in Dadonaite et al. 2024 (21). See

bioRxiv preprint doi: https://doi.org/10.1101/2025.01.17.633612; this version posted January 20, 2025. The copyright holder for this preprint (which was not certified by peer review) is the author/funder, who has granted bioRxiv a license to display the preprint in perpetuity. It is made available under aCC-BY 4.0 International license.

https://dms-vep.org/SARS-CoV-2_XBB.1.5_spike_DMS_infant_sera/individual.html for interactive versions of these plots for all four sera sets.

bioRxiv preprint doi: https://doi.org/10.1101/2025.01.17.633612; this version posted January 20, 2025. The copyright holder for this preprint (which was not certified by peer review) is the author/funder, who has granted bioRxiv a license to display the preprint in perpetuity. It is made available under aCC-BY 4.0 International license.

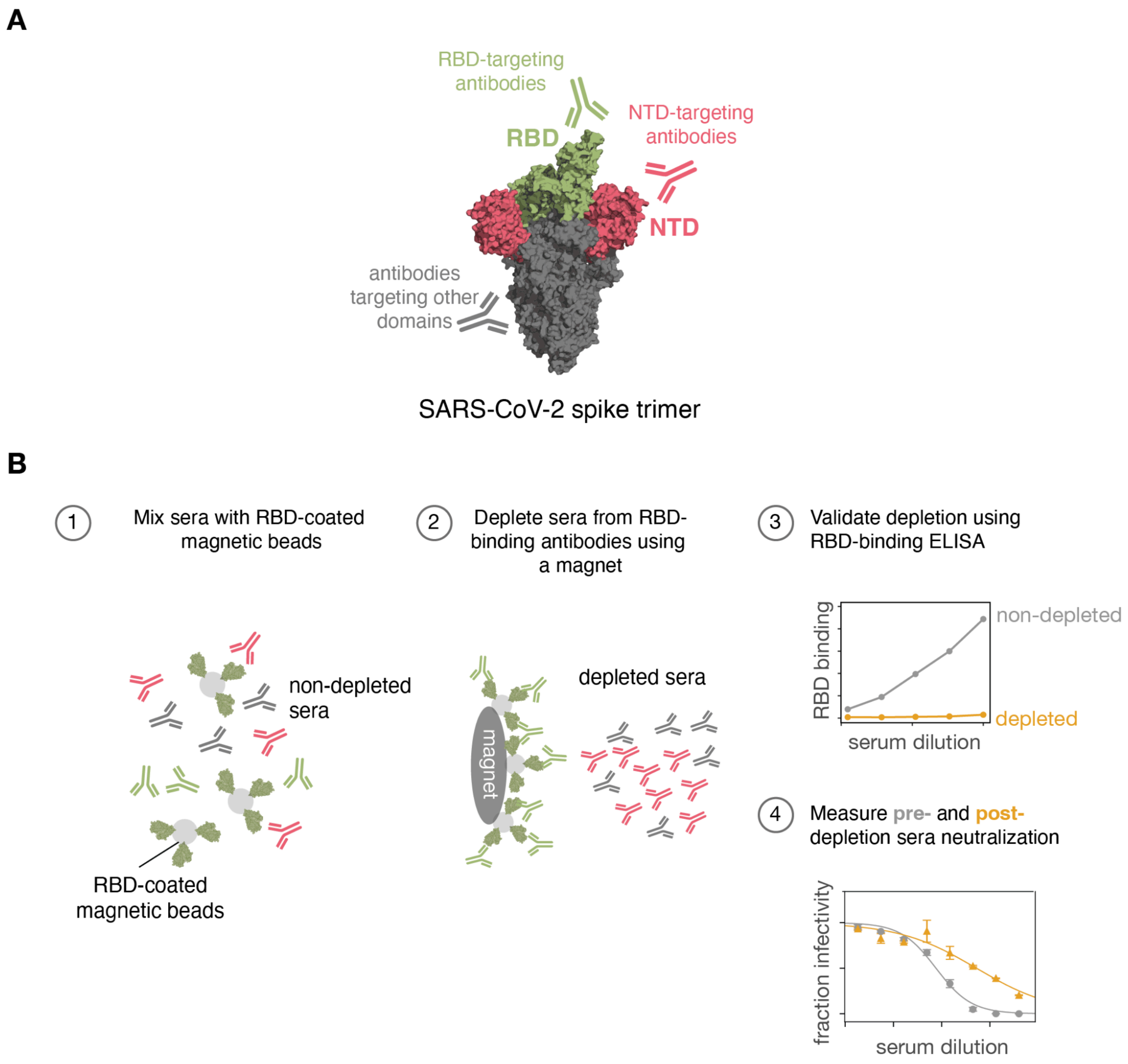

Supplementary Figure 3. Depletion of RBD-binding antibodies from sera to measure their contribution to neutralization

A. Schematic of SARS-CoV-2 spike trimer labeling different domains (PDB: 6XM4). **B.** Each serum was mixed with RBD coated magnetic beads to allow RBD-targeting antibodies to bind the beads. After binding, a magnet was used to deplete sera of the beads. To validate successful RBD-binding antibody depletion enzyme-linked immunosorbent assay (ELISA) was used. Depleted and non-depleted sera was used to perform pseudovirus neutralization assays.

bioRxiv preprint doi: https://doi.org/10.1101/2025.01.17.633612; this version posted January 20, 2025. The copyright holder for this preprint (which was not certified by peer review) is the author/funder, who has granted bioRxiv a license to display the preprint in perpetuity. It is made available under aCC-BY 4.0 International license.

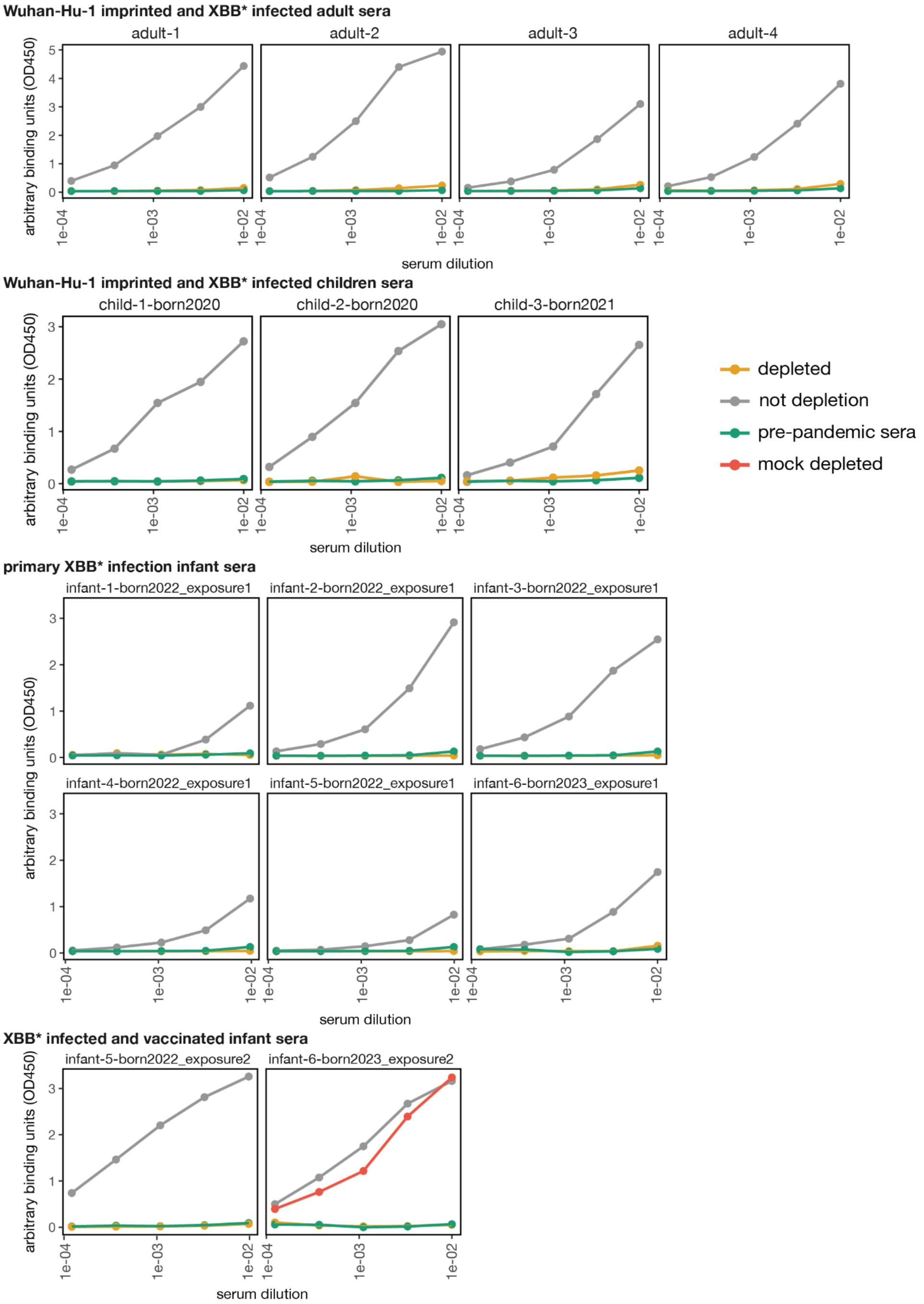

Supplementary Figure 4. Binding of sera to RBD-coated ELISA plates pre- and post-depletion of RBD-binding antibodies

Depleted and non-depleted sera binding to XBB.1.5 RBD-coated plates measured using enzyme-linked immunosorbent assay (ELISA). Serum depletion was performed as described in **Fig. S2**. Sera was depleted by multiple rounds of XBB.1.5 RBD-coated bead incubation until its binding was similar to the pre-pandemic sera (sera collected prior to 2020 that should not contain any antibodies specific to SARS-CoV-2). A mock depleted sample, which was incubated with beads lacking XBB.1.5 RBD, is shown for *infant-6-born2020_exposure2* serum.

bioRxiv preprint doi: https://doi.org/10.1101/2025.01.17.633612; this version posted January 20, 2025. The copyright holder for this preprint (which was not certified by peer review) is the author/funder, who has granted bioRxiv a license to display the preprint in perpetuity. It is made available under aCC-BY 4.0 International license.

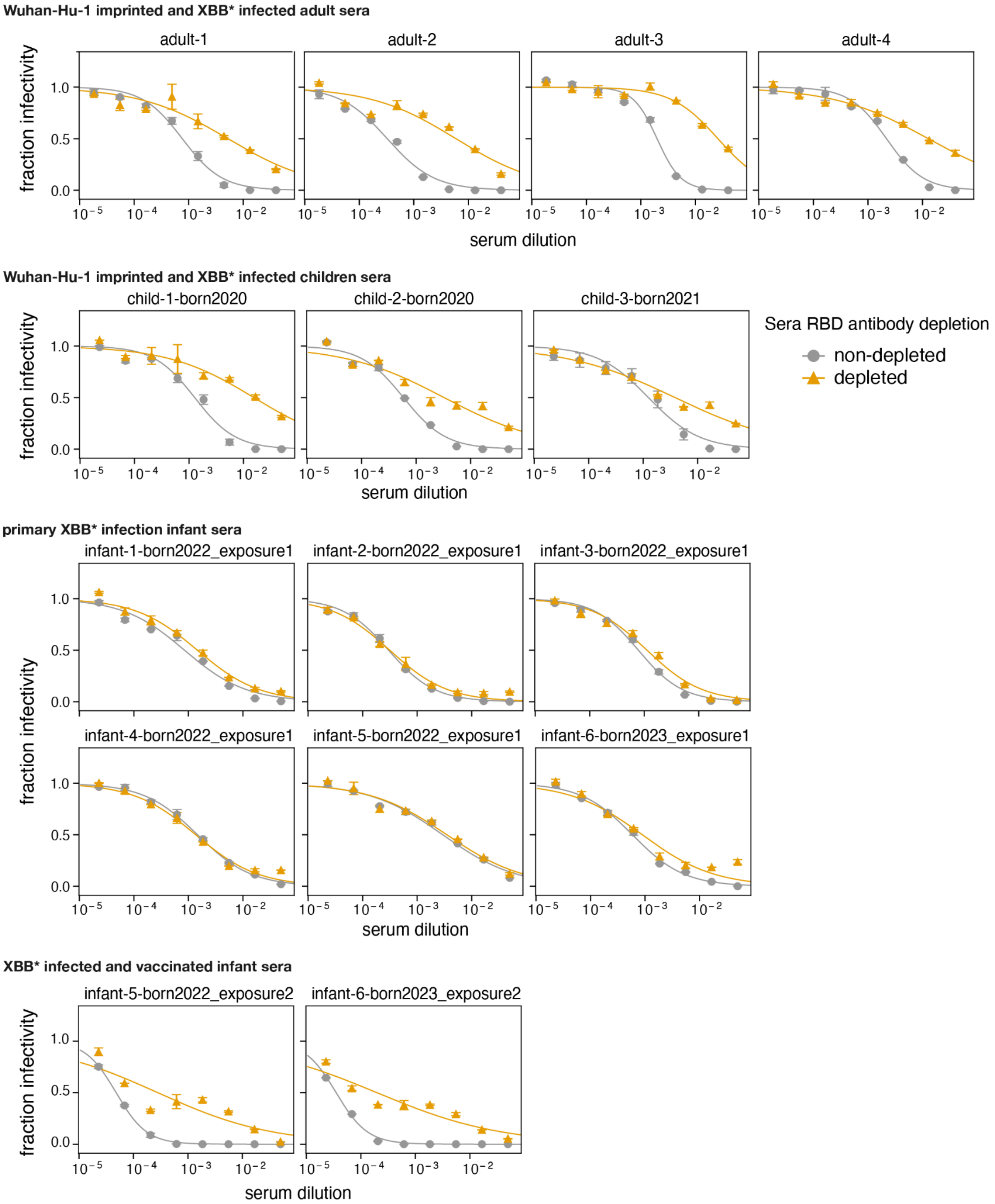

Supplementary Figure 5. Neutralization of XBB.1.5 pseudovirus pre- and post-depletion of RBD-binding antibodies

Neutralization curves for XBB.1.5 pseudovirus against depleted and non-depleted sera from **Fig. S3**. Neutralization assays were performed on 293T cells expressing medium levels of ACE2 as described in Farrell et al. 2022 (20). These target cells enable better detection of the neutralizing activity NTD-binding antibodies than standard 293T-ACE2 cells that express higher levels of ACE2.

bioRxiv preprint doi: https://doi.org/10.1101/2025.01.17.633612; this version posted January 20, 2025. The copyright holder for this preprint (which was not certified by peer review) is the author/funder, who has granted bioRxiv a license to display the preprint in perpetuity. It is made available under aCC-BY 4.0 International license.

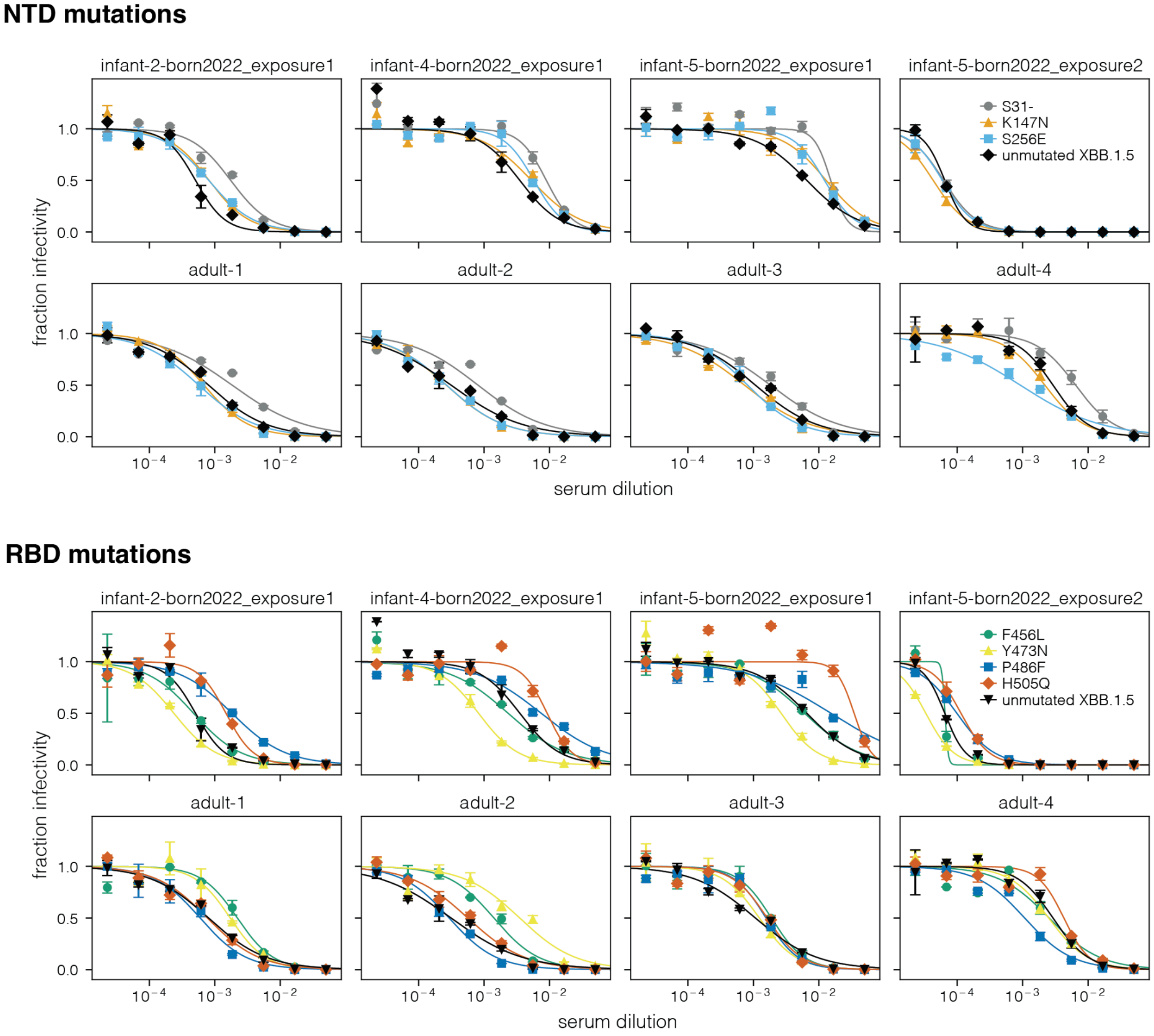

Supplementary Figure 6. Pseudovirus neutralization of different XBB.1.5 spike mutants

Neutralization curves for unmutated XBB.1.5 and mutant XBB.1.5 spike pseudoviruses against different sera. All mutants contain a single amino-acid change on the background of the XBB.1.5 spike.

bioRxiv preprint doi: https://doi.org/10.1101/2025.01.17.633612; this version posted January 20, 2025. The copyright holder for this preprint (which was not certified by peer review) is the author/funder, who has granted bioRxiv a license to display the preprint in perpetuity. It is made available under aCC-BY 4.0 International license.

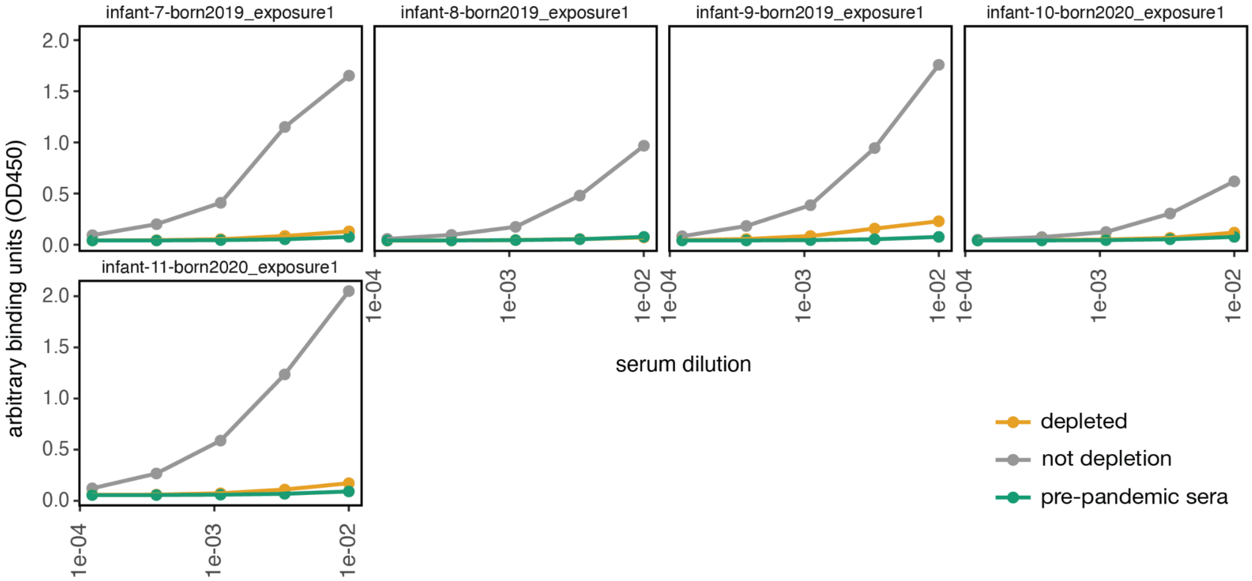

Supplementary Figure 7. Binding of sera to RBD-coated ELISA plates pre- and post-depletion of RBD-binding antibodies for primary Wuhan-Hu-1 infant infection sera
Depleted and non-depleted sera binding to Wuhan-Hu-1 RBD-coated plates measured using enzyme-linked immunosorbent assay (ELISA). Serum depletion was performed as described in **Fig. S2** except using beads conjugated to the Wuhan-Hu-1 RBD rather than the XBB.1.5 RBD. Sera was depleted in multiple rounds of RBD-coated bead incubation until its binding was similar to the pre-pandemic sera (sera collected prior to 2020 that should not contain any antibodies specific to SARS-CoV-2). Note that depletions for *primary Wuhan-Hu-1 infection adult sera* were done for a previous study and that same pre- and post-depletion sera was reused for this study; pre- and post-depletion ELISA binding data for that sera comparable to that shown here can be found in the associated Greaney et al. (2021) paper (13).

bioRxiv preprint doi: https://doi.org/10.1101/2025.01.17.633612; this version posted January 20, 2025. The copyright holder for this preprint (which was not certified by peer review) is the author/funder, who has granted bioRxiv a license to display the preprint in perpetuity. It is made available under aCC-BY 4.0 International license.

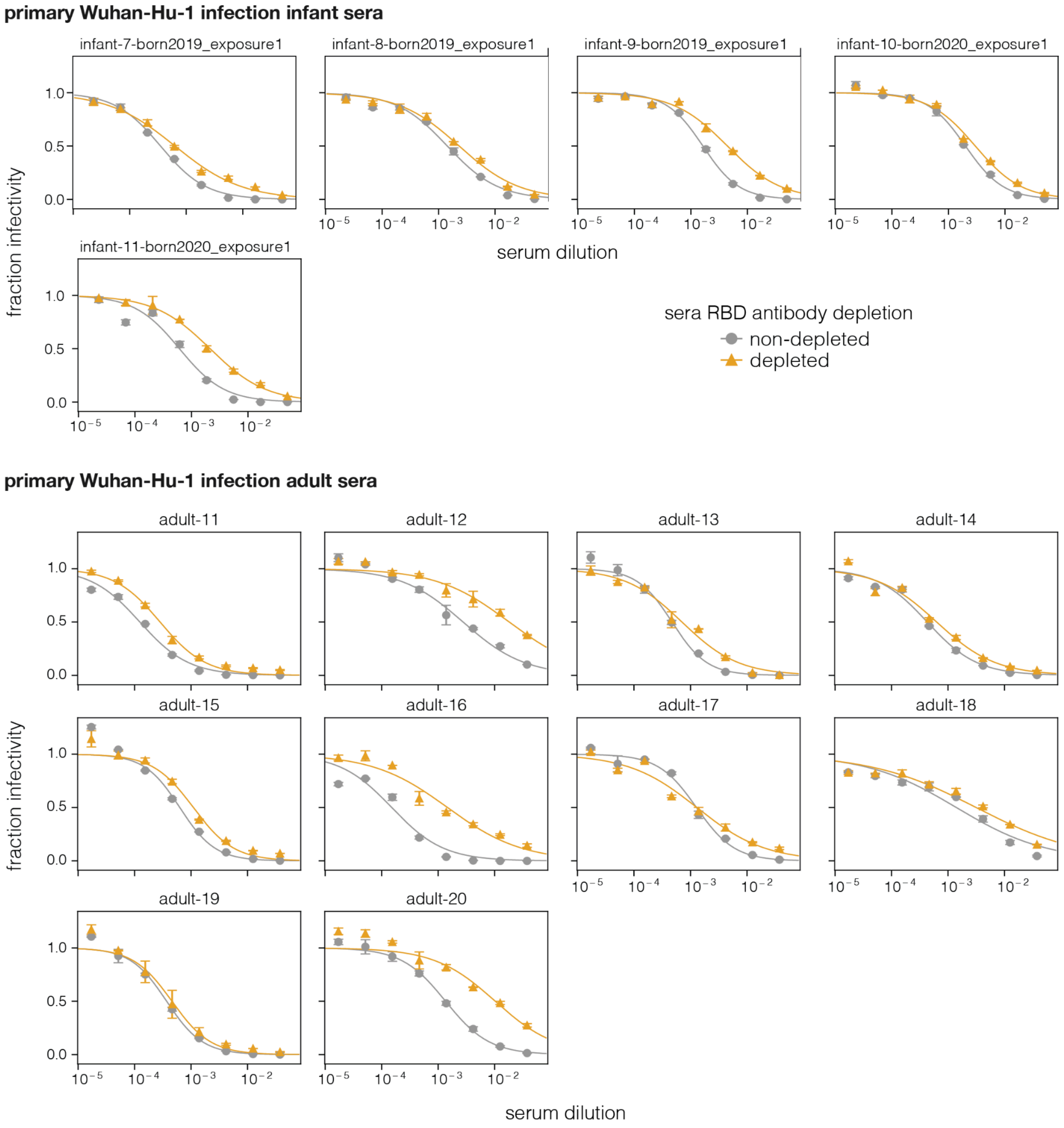

Supplementary Figure 8. Neutralization of D614G pseudovirus pre- and post-depletion of RBD-binding antibodies for primary Wuhan-Hu-1 infections

Neutralization curves for D614G pseudovirus against depleted and non-depleted primary Wuhan-Hu-1 infection infant and adult sera. Neutralization assays were performed on 293T cells expressing medium levels of ACE2 cells as described in Farrell et al. 2022 (38).

bioRxiv preprint doi: https://doi.org/10.1101/2025.01.17.633612; this version posted January 20, 2025. The copyright holder for this preprint (which was not certified by peer review) is the author/funder, who has granted bioRxiv a license to display the preprint in perpetuity. It is made available under aCC-BY 4.0 International license.

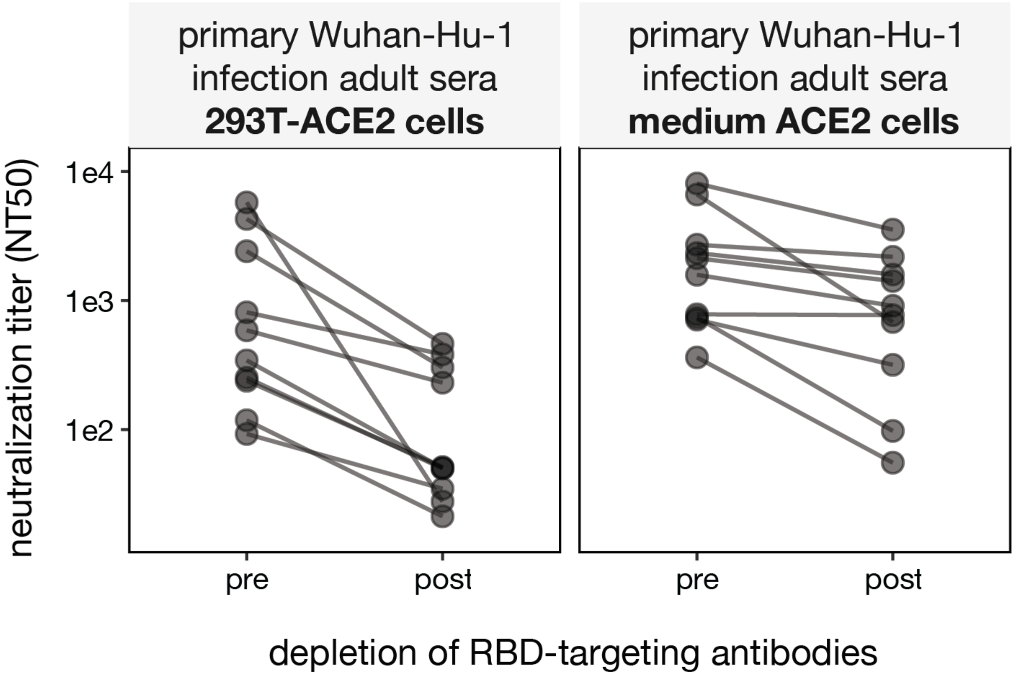

Supplementary Figure 9. The extent that serum neutralization is due to RBD-binding antibodies depends on the ACE2 expression of the target cells

Neutralization of pseudovirus expressing the D614G spike by *primary Wuhan-Hu-1 infection adult sera* pre- and post-depletion of Wuhan-Hu-1 RBD targeting antibodies as measured on two different target cell lines. The left panel shows data replotted from Greaney et al. 2021 (13) for neutralization assays performed using the 293T-ACE2 cells (37), which express high levels of ACE2. The right panel shows new measurements made using the same pre- and post-depletion serum samples but with the 293T cells expressing medium levels of ACE2 (38). These medium ACE2 cells express less ACE2 than the 293T-ACE2 cells; see first supplementary figure of Farrell et al. 2022 (38).

bioRxiv preprint doi: https://doi.org/10.1101/2025.01.17.633612; this version posted January 20, 2025. The copyright holder for this preprint (which was not certified by peer review) is the author/funder, who has granted bioRxiv a license to display the preprint in perpetuity. It is made available under aCC-BY 4.0 International license.

Supplementary Tables

https://docs.google.com/spreadsheets/d/1CfmnPS4G_eMFGGlSMzhalu7v0CDxQAgNAmP7YlPIRmk/edit?gid=0#gid=0

Supplementary Table 1. Details of the individual sera used in this study

Individual sera from each cohort used in this study. *Primary Wuhan-Hu-1 infected adult sera* and some of the *Wuhan-Hu-1 imprinted and XBB* infected adult sera* do not have sequencing data available for infections, however, based on infection dates the former cohort is most likely exposed to early strains closely related to Wuhan-Hu-1, and the latter to XBB*-related variants. The nasal swab from *infant-6-born2023_exposure1* did not yield sufficient coverage to confirm infecting variant but based on the date exposed this was most likely XBB* related variant. Negative values in the days post-exposure column indicate the number of days before exposure the sera were collected.
